# Differential Susceptibility and Response of Primary Human Myeloid BDCA1^+^ Dendritic Cells to Infection with Different Enteroviruses

**DOI:** 10.1371/journal.pone.0062502

**Published:** 2013-04-24

**Authors:** Barbara M. Schulte, Esther D. Kers-Rebel, Amy C. Prosser, Jochem M. D. Galama, Frank J. M. van Kuppeveld, Gosse J. Adema

**Affiliations:** 1 Department of Tumor Immunology, Nijmegen Centre for Molecular Life Sciences & Radboud University Nijmegen Medical Centre, Nijmegen, The Netherlands; 2 Department of Medical Microbiology, Nijmegen Centre for Molecular Life Sciences & Radboud University Nijmegen Medical Centre, Nijmegen, The Netherlands; Leiden University Medical Center, The Netherlands

## Abstract

Coxsackie B viruses (CVBs) and echoviruses (EVs) form the Human Enterovirus-B (HEV-B) species within the family Picornaviridae. HEV-B infections are widespread and generally cause mild disease; however, severe infections occur and HEV-B are associated with various chronic diseases such as cardiomyopathy and type 1 diabetes. Dendritic cells (DCs) are the professional antigen-presenting cells of our immune system and initiate and control immune responses to invading pathogens, yet also maintain tolerance to self-antigens. We previously reported that EVs, but not CVBs, can productively infect *in vitro* generated monocyte-derived DCs. The interactions between HEV-B and human myeloid DCs (mDCs) freshly isolated from blood, however, remain unknown. Here, we studied the susceptibility and responses of BDCA1^+^ mDC to HEV-B species and found that these mDC are susceptible to EV, but not CVB infection. Productive EV7 infection resulted in massive, rapid cell death without DC activation. Contrary, EV1 infection, which resulted in lower virus input at the same MOI, resulted in DC activation as observed by production of type I interferon-stimulated genes (ISGs), upregulation of co-stimulatory and co-inhibitory molecules (CD80, CD86, PDL1) and production of IL-6 and TNF-α, with a relative moderate decrease in cell viability. EV1-induced ISG expression depended on virus replication. CVB infection did not affect DC viability and resulted in poor induction of ISGs and CD80 induction in part of the donors. These data show for the first time the interaction between HEV-B species and BDCA1^+^ mDCs isolated freshly from blood. Our data indicate that different HEV-B species can influence DC homeostasis in various ways, possibly contributing to HEV-B associated pathology.

## Introduction

Dendritic cells (DCs) are the professional antigen-presenting cells of the immune system that are key players in initiating and modulating innate and adaptive immune responses as well as in maintaining tolerance. DCs express a variety of pattern recognition receptors (PRRs), such as Toll-like receptors (TLRs) and RIG-I-like receptors (RLRs), which they use to recognize pathogens, pathogen-associated molecules, or pathogen induced-damage [1], [2]. For example, TLR3 and the RLRs RIG-I (retinoic acid inducible gene I) and Mda5 (melanoma differentiation- associated gene 5) are PRRs involved in sensing double-stranded (ds) viral RNA. Triggering of PRRs results in phenotypic maturation of the DC and production of pro-inflammatory cytokines, enabling the DC to initiate antiviral responses [3]. DCs (cross)present viral peptides to CD4^+^ T cells and CD8^+^ T cells to eliminate virus infected cells [4], [5]. Viruses, however, have co-evolved with their hosts and evade antiviral immune responses via several ways. Some viruses, for example, are known to infect DCs directly, interfere with their function, and thereby hamper antiviral responses [6], [7], [8], [9].

Members of the human enterovirus B (HEV-B) species of the Picornaviridae family, such as coxsackie B viruses (CVB) and echoviruses (EV) are small, non-enveloped, single-stranded RNA viruses with a lytic life cycle. Most infections of these widespread viruses remain limited to the gastrointestinal tract. However, during more severe infections also secondary target organs such as the heart, pancreas and brain may be infected, resulting in e.g. myocarditis, pancreatitis or (meningo)encephalitis [10]. Additionally, HEV-B infections have been associated with development of autoimmune diseases such as type 1 diabetes (T1D) [11], [12], [13], [14]. We have previously studied susceptibility and responses of *in vitro* generated monocyte-derived DCs (moDCs) to HEV-B [9]. CVBs were unable to directly infect moDC, probably due to lack of the viral entry receptor CAR (Coxsackie- and Adenovirus Receptor) [9]. EVs did successfully infect moDCs. One EV (EV9 Hill) was studied in more detail and we reported that productive infection with EV9 Hill did not result in DC activation (e.g. upregulation of costimulatory molecules and pro-inflammatory cytokine production). Instead, infection resulted in rapid loss of responsiveness to TLR ligands and cell death. These findings suggest that EVs can interfere with immune homeostasis via direct infection of DCs.

Our previous studies were performed with moDCs which are *in vitro* differentiated from monocytes by addition of IL-4 and GM-CSF. Due to the low frequency of naturally occurring DCs in blood (<1% of PBMCs), many studies on DC function and biology are performed with these moDCs. However, studying naturally occurring myeloid DCs (mDCs) derived freshly from blood may result in a different outcome. Naturally occurring mDCs, have unique gene expression profiles distinct from moDC, suggesting they can perform different functions [1], [15], [16], [17]. Indeed differences in biological functions e.g. antigen presentation capacity and cytokine production have been reported [18], [19], [20], thus studies that investigate these naturally occurring mDCs are warranted. Moreover, multiple studies have found enteroviral RNA in blood and PBMCs of T1D patients, yet the source for viral RNA as well as its role in T1D pathogenesis remains to be established. Direct infection of blood DCs could potentially play a role e.g. by (chronic) immune activation. Currently, no studies on the susceptibility to, and response of, human primary blood mDCs to HEV-B have been published, probably because of the low frequency of these DCs in blood, which makes such studies more challenging compared to studies with moDCs. Two subsets of naturally occurring mDCs have been described, BDCA1^+^ (CD1c^+^) mDCs and BDCA3^+^ (CD141^+^) mDCs [15], [21], [22]. Both subsets have specialized functions that are only beginning to be unraveled, for example they differ in their capacity to phagocytose pathogens, produce cytokines and stimulate T cells [15], [16], [17], [23], [24]. In this study, we set out to investigate susceptibility and responses of the most abundant human mDC subset, BDCA1^+^ mDCs, freshly isolated from blood, to HEV-B.

We show that primary BDCA1^+^ mDC can be efficiently infected with various EVs, yet not by CVBs. Productive EV infection of human BDCA1^+^ mDC isolated freshly from blood results in ***a)*** induction of innate type I interferon (IFN) responses and increased CD80 and PDL1 expression *or* ***b)*** rapid cell death without apparent induction of type I IFNs or maturation, depending on the efficiency of EV-infection. Our results demonstrate that different HEV-B viruses have different tropism for human BDCA1^+^ mDCs and that productive EV-infection has different outcome depending on the amount of virus capable to infect DCs. This may have significant implications for HEV-B pathogenesis, e.g. inefficient clearance of the virus when DCs are killed and induction of adaptive anti-viral responses is hampered.

## Materials and Methods

### Virus Stocks and Purification

Reference strains Echovirus 1 Farouk (EV1), EV7 Wallace (EV7), EV8 Bryson (EV8), EV9 Hill (EV9) and EV11 Gregory (EV11) were obtained from the National Institute for Public Health and the Environment (RIVM, Bilthoven, The Netherlands). CVB3 Nancy (CVB3) and CVB4 E2 were kindly provided by R. Kandolf (University of Tübingen, Germany) and Y.W. Yoon (University of Calgary, Canada). Production of virus stocks and virus titrations were performed on buffalo green monkey cells as described previously. Serial 10-fold dilutions were tested in 96-well microtiter plates and 50% Tissue Culture Infective Doses (TCID_50_) were calculated as described before [9].

### Isolation, Culture and Infection of Cells

DCs were isolated from buffy coats from peripheral blood of anonymous healthy blood donors which were obtained from Sanquin bloodbank, Nijmegen according to institutional guidelines and the declaration of Helsinki. For the experiments described herein a total of 21 buffy coats from healthy donor were used. Myeloid BDCA1^+^ DCs (mDCs) were isolated using BDCA-1 beads, using the CD1c^+^ DC isolation kit (Miltenyi Biotec). Cells were routinely up to 95% pure, as assessed by double staining for BDCA-1/CD11c (Miltenyi Biotec, and BD Pharmingen, respectively). Immediately after isolation cells were used for infection experiments. Monocyte-derived DCs (moDCs) were generated and infected as described previously [9]. BDCA1^+^ mDCs were infected with indicated viruses at indicated multiplicity of infection (MOI) in X-Vivo 15 (Lonza). After 1 hour incubation at 37°C, cells were thoroughly washed and plated out in X-Vivo supplemented with 10% FCS and 400 U/ml GM-CSF (Strahtman). For replication experiments an input sample was taken at t = 0, i.e. after the infection period and the washes, and at indicated times after infection. Virus presence was determined in cells plus supernatant combined by endpoint titration as described above. In some experiments 50 µM rupintrivir (AG7088, a kind gift from Pfizer) was added directly after infection. Poly I:C (polyinosinic-polycytidylic acid) was from Enzo life sciences and was used at 20 µg/ml.

### RNA Isolation and Quantitative PCR (qPCR)

RNA isolations were done using the ZR RNA isolation kit (Zymo Research) according to manufacturer’s instructions. RNA was treated with DNase I (amplification grade; Invitrogen) and reverse-transcribed into cDNA by using random hexamers and Moloney murine leukemia virus reverse transcriptase (Invitrogen). To exclude genomic DNA contamination we included a “−RT” control in which the reverse transcriptase was replaced with RNase-free water. The “−RT” control was taken along in the qPCR analysis. cDNA was stored at −20°C until further use. mRNA levels for the genes of interest were determined by quantitative PCR (qPCR) with a Biorad CFX apparatus (Biorad) with SYBR Green (Applied Biosystems). Analysis was done using Biorad CFX-1.6 software mRNA levels of the genes of interest were normalized to mRNA levels of the housekeeping gene HPRT (Hypoxanthine-guanine phosphoribosyltransferase) or GAPDH (Glyceraldehyde 3-phosphate dehydrogenase) as indicated and were calculated according to the cycle threshold method [25]. As a cut-off for reliable PCR analysis we used 35 Ct cycles. Primer sequences are available on request and were from the Primer Bank database [26].

### Flow Cytometry

Cells were harvested with cold PBS at indicated time points, washed twice in PBA (PBS containing 0.5% bovine serum albumin and 0.01% sodium azide) with 2% human serum. Cells were incubated with conjugated cell surface markers or corresponding isotypes for 20 minutes on ice followed by 2 washing steps in PBA. CD55, CD80, CD86, PD-L1 were from BD Pharmingen. CD49b (26G8 clone) was a kind gift from dr. de Fougerolles (Biogen) and hCAR (RcmB clone) was a gift from dr. J Bergelson (University of Pennsylvania, USA). Expression of costimulatory molecules was analysed on CD11c^high^ expressing gated mDCs. For intracellular staining cells were washed in PBS, fixed using 2% paraformaldehyde for 4 minutes on ice, washed in PBA and permeabilized using 0.1% saponin in PBA. Cells were subsequently blocked using PBA/0.1% saponin/2% human serum for 30 minutes on ice followed by incubation with the J2 double-stranded RNA antibody or corresponding isotype (English & Scientific Consulting Bt.) in PBA/0.1% saponin for 30 minutes on ice, washed, and incubated with an Alexa-488 conjugated rabbit-anti-mouse IgG2a in PBA/0.1% saponin for 30 minutes on ice. Viability was analyzed using AnnexinV (BD Pharmingen) and 7-AAD (eBioscience) staining. Cells were analysed on a CyAn Flow cytometer (Beckman Coulter) and data was analysed using Summit software and FlowJo. Gating strategies included exclusion of dead cells and debris in forward/side scatter. Mean fluorescence intensity values are shown for the entire population of DCs.

### Western Blot

Western blotting was performed as described [27]. Anti-Mda5 and anti-3D polymerase antibodies were generous gifts from dr. P.B. Fisher (Virginia Commonwealth University, School of Medicine, Richmond, USA) and dr. C. Cameron (Pennsylvania State University, USA), respectively, and were used in a 1∶10000 and 1∶1000 dilution. Quantification of intensity relative to actin was calculated using Odyssey 2.1 software and is calculated as (intensity protein X/intensity actin) * 1000.

### ELISA

TNF-α and IL-6 production was analyzed with the human TNF ELISA kit (BD Biosciences) and PeliPair human IL-6 Elisa (Sanquin, Amsterdam, The Netherlands) respectively, according to manufacturer’s instruction.

### Statistical Analysis

Statistical analysis was performed using Students T-test (2 tailed distribution) or ANOVA followed by post-hoc Tukey test, as indicated. A p-value <0.05 was considered a significant difference.

## Results

### EVs, but Not CVBs Replicate in Primary Human BDCA1^+^ mDCs and Induce Cell Death

**Figure 1:**
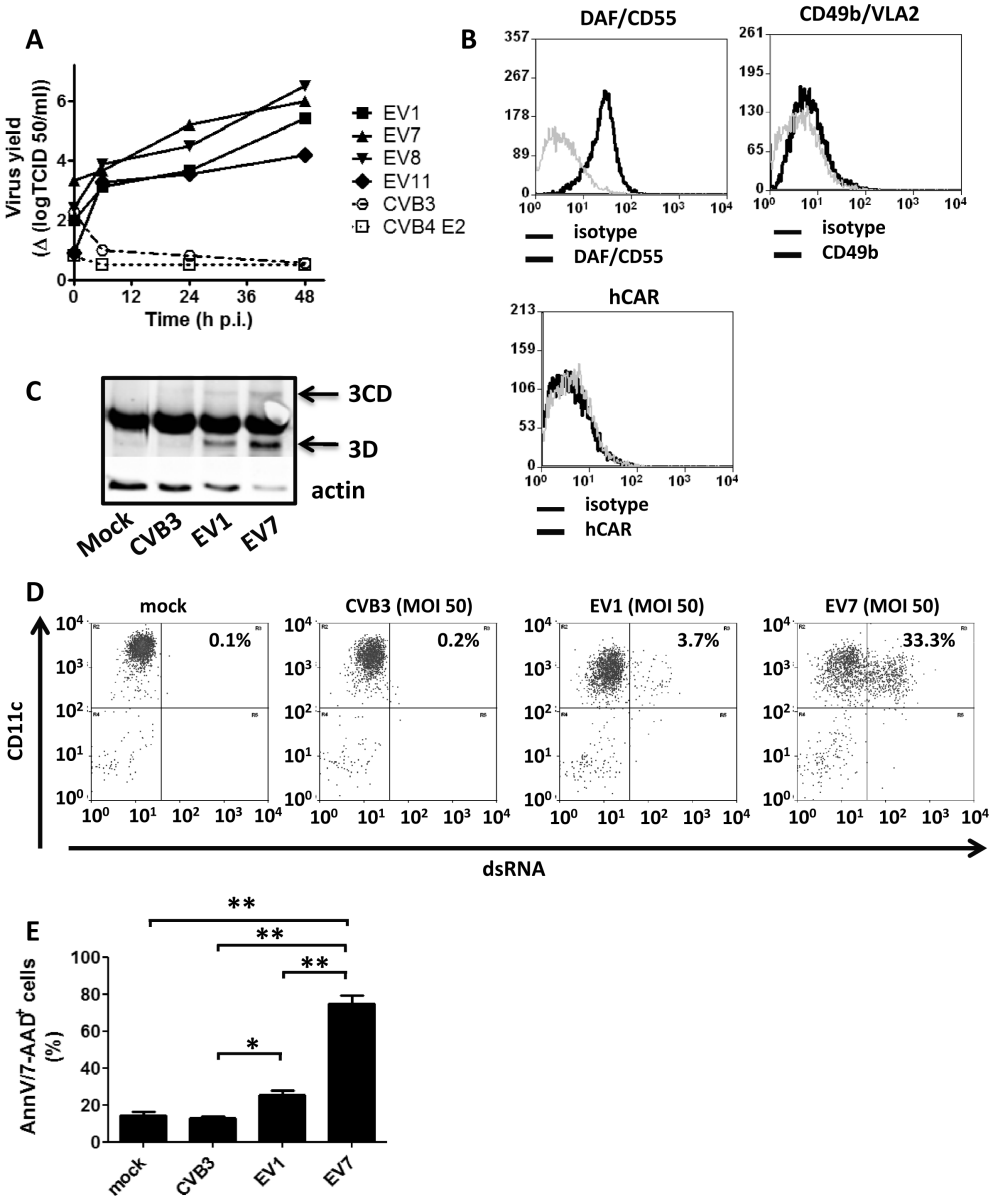
Replication of human enteroviruses in primary human myeloid BDCA1^+^ dendritic cells. A) Freshly isolated BDCA1^+^ mDCs were infected with indicated viruses at an MOI of 2 and replication was assessed at indicated time points by endpoint titration. B) Freshly isolated mDCs were stained with indicated antibodies or corresponding isotypes and analyzed using flowcytometry. C) mDCs were infected as indicated (MOI 50) and after 18 h infection 3D polymerase protein expression was assessed by western blot analysis. D) mDCs infected as indicated were harvested 18 h after infection using cold PBS and amount of dsRNA was analyzed using flowcytometry. E) mDCs infected as in D) were tested for cell viability using Annexin V/7-AAD double staining. Statistical significance was determined using ANOVA and post-hoc Tukey test. Shown are representative experiments of more than 3 independent experiments using different donors (A–D) or average of 3 independent experiment (E) (mean values+ SEM). * p<0.05; ** p<0.001.

To investigate the susceptibility of highly purified (>90%) blood BDCA1^+^ mDCs to HEV-B, they were exposed to a series of EVs (EV1 Farouk, EV7 Wallace, EV8 Bryson, EV9 Hill and EV11 Gregory) and CVBs (CVB3 Nancy and CVB4 E2) followed by replication analysis. EV1, EV7, EV8, and EV11 were all able to replicate in BDCA1^+^ mDCs, whereas CVB3 and CVB4 were unable to yield infectious virus (**Fig. 1A**). EV9 Hill replicated in BDCA1^+^ mDCs from only 50% of the donors (**Fig. S1**), whereas this EV strain efficiently infects and rapidly kills moDCs (11). The reason for this may be that the virus entry receptor for EV9 Hill is not present on BDCA1^+^ mDCs isolated freshly from blood from some donors, but is present on moDCs; however, since the receptor used by EV9 Hill is still matter of debate (16, 18) we were unable to determine receptor expression levels of the EV9 Hill entry receptor. Expression of the virus entry receptor for EV7 and EV11 (DAF, CD55) was high on mDC, whereas the EV1 and EV8 entry receptor (VLA2, CD49b) was expressed at very low levels (**Fig. 1B**). Consistent with higher DAF expression, we found higher binding of EV7 to BDCA1^+^ mDCs directly after infection (**Fig. S2**). Yield of infectious virus after 48 h did, however, not differ between EV1 and EV7 infection. In BGM cells, that have high expression of both DAF and VLA2 (data not shown) we did not observe differences in input or virus yield (**Fig. S3**). Absence of replication of CVBs can be explained by lack of the CAR receptor on BDCA1^+^ mDCs, whereas CAR is readily detected on HEK cells and BGM cells (**Fig. 1B** **and data not shown**).

Infection of BDCA1^+^ mDCs was confirmed by western blot analysis. Viral 3D polymerase and its 3CD precursor were detected in EV1- and EV7-infected BDCA1^+^ mDC protein lysates, whereas this was not the case for CVB3 or mock-infection (**Fig. 1C**). To confirm that indeed the BDCA1^+^ mDCs were the infected cells and not contaminating other leukocytes, such as B or T lymphocytes, we analyzed DCs by staining for the DC marker CD11c and double-stranded RNA (dsRNA) using flowcytometry. The J2 anti-dsRNA Ab detects dsRNA which is produced by many RNA viruses, including enteroviruses, as an intermediate during their replication cycle [28]. Anti-dsRNA staining of CVB-infected BGM cells shows a good correlation with MOI (**Fig. S4**). As shown in **Fig. 1D** dsRNA positive cells cannot be detected in mock-infected or CVB3 infected BDCA1^+^/CD11c^high^ mDCs. In contrast, dsRNA staining is readily detected in EV1 and EV7-infected BDCA1^+^/CD11c^high^ mDC. We further confirmed that there was no increase in virus titer upon infection of isolated B-cells, which are the main contaminating cells in the BDCA1^+^ mDC population (**Fig. S5**), corroborating that virus replication indeed occurs in the BDCA1^+^ myeloid DC population. Notably, we detected higher amounts of 3D protein and dsRNA positive cells upon EV7 infection compared to EV1 infection. Thus it seems that EV1 less efficiently binds and/or enters the BDCA1^+^ mDCs and that less viral proteins and dsRNA is produced upon EV1 infection.

Virus release upon enterovirus infection occurs via cell lysis, thus we examined cell viability in DCs upon infection. As expected, CVB3 infection did not result in any increased cell death. EV1 infection resulted in a modest increase in cell death 20 h p.i. whereas upon EV7 infection rapid cell death was observed (**Fig. 1E** **and Fig. S6**). The more pronounced cell death upon EV7 infection correlated with higher 3D viral polymerase expression and detection of more dsRNA positive cells compared to EV1 (**Fig. 1C and 1D**). These data thus demonstrate that CVBs cannot infect BDCA1^+^ mDCs isolated freshly from blood, whereas EVs can; yet differences to induce cell death exist among the EV stains, and correlate with the amount of virus that binds and/or enters the cells after infection (**Fig. S2**).

### BDCA1^+^ mDCs Induce Antiviral IFN-α/β Responses upon Infection with EV1

**Figure 2:**
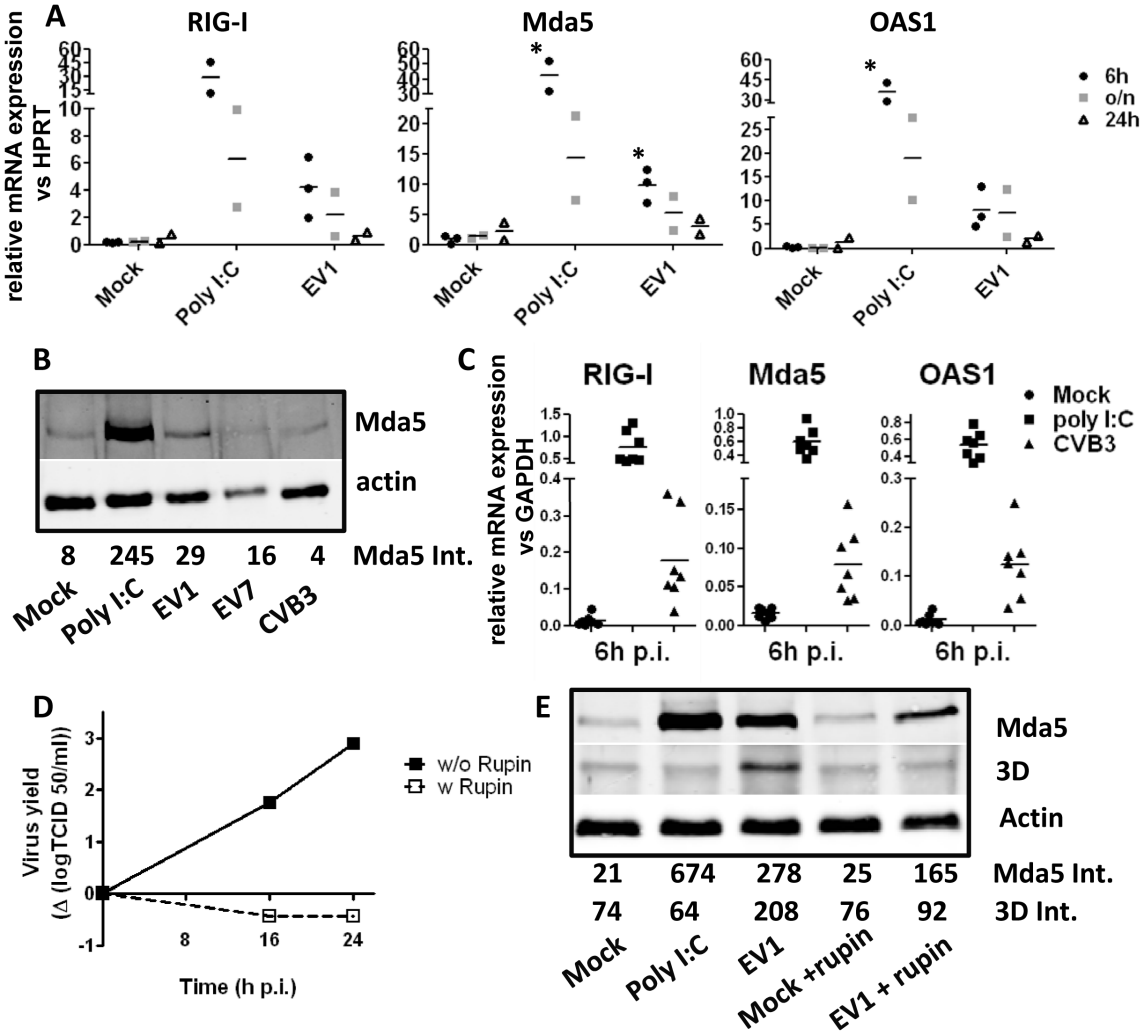
ISG induction in HEV-B infected primary human myeloid BDCA1^+^ dendritic cells is dependent on virus replication. A) Freshly isolated BDCA1^+^ mDCs were infected (EV1, MOI 50), stimulated with poly I:C (20 µg/ml) or left untreated and at indicated times RNA was isolated and qPCR was performed as described. Statistical significance versus mock-infected cells determined by Students T-test, *P<0.05. B) mDCs were infected with indicated viruses (MOI 50) or stimulated as for A) and after 18 h protein expression was assessed by western blot analysis. The intensity given below the image (Mda5 Int.) represents Mda5 intensity relative to actin calculated as described in materials & methods section. C) Freshly isolated BDCA1^+^ mDCs were infected (CVB3, MOI 50), stimulated with poly I:C (20 µg/ml) or left untreated and at 6 h p.i. RNA was isolated and qPCR was performed as described. D) mDCs were infected with EV1 (MOI 50), washed in PBS and plated out. Directly after infection rupintrivir (50 µM) (indicated as w Rupin) was added and replication was analyzed as for Fig. 1A) E) cells were infected as in D), stimulated with polyI:C (20 µg/ml) or left unstimulated and after 20 h protein expression was analyzed by western blot. The intensity given below the image represents Mda5 or 3D intensity relative to actin calculated as described in materials & methods section.+rupin indicates that rupintrivir was added to the DC cultures directly after plating the cells following infection. Shown are representative experiments of 3 (B), individual donor results+average from 3 (A) or 7 (C), or representative of 2 (D, E) independent experiments using different donors.

Type I interferons (IFN-α/β) are produced and secreted upon virus infection and induce interferon-stimulated genes (ISGs) which encode proteins that aid in detection of viruses or function as effector molecules important for virus elimination. The ISGs RIG-I and Mda5 are RIG-like receptors that are involved in recognition of a variety of RNA viruses [29], [30]. One of the effector proteins is oligo-adenylate synthetase 1 (OAS1) that activates RNaseL which subsequently degrades RNA and prevents further virus replication. Using qPCR we assessed whether these ISGs were induced upon infection with HEV-B. Poly I:C (20 µg/ml) was used as a positive control. As shown in **Fig. 2A** ISGs were detected at 6 h p.i. in EV1-infected BDCA1^+^ mDC. This induction was transient and after overnight incubation mRNA expression had decreased to nearly basal levels (**Fig. 2A**). Western Blot analysis 18 h p.i. revealed that EV1 also induced Mda5 and RIG-I protein expression upon infection (**Fig. 2B** **and Fig. S8**). In contrast to EV1, EV7 infection induced only modest ISG levels at mRNA level (**Fig. S7**) and did not result in induction of ISGs at the protein level (**Fig. 2B** **and Fig. S8**) suggesting that the rapid induction of cell death described above prevents production of ISGs at the protein level in EV7-infected BDCA1^+^ DCs. Upon CVB3 infection ISGs were induced at mRNA level in most donors, but interestingly, in some donors (approximately 30%) we did not find clear induction of ISGs (i.e. induction of >5-fold expression relative to mock-infected cells)(**Fig. 2C**). The reason for this finding is currently unknown. In contrast, at the protein level Mda5 induction was absent in most donors upon CVB-infection (**Fig. 2B**), or induced at very low levels (data not shown).

To assess whether virus replication is required for the induction of ISGs upon EV1 infection we analyzed ISG induction in the presence of rupintrivir, a known inhibitor of enterovirus replication. EV1 replication was efficiently inhibited by rupintrivir indicated by absence of virus titer increase upon infection (**Fig. 2D**) and a near complete absence of 3D polymerase (**Fig. 2E**) in the presence of this drug. EV1-induced expression of Mda5 was decreased 4-fold when rupintrivir was added directly after infection (**Fig. 2E**), indicating that virus replication is at least partially required for efficient ISG induction. Induction of ISGs was not completely abrogated, indicating that virus replication-independent mechanisms can also induce ISGs, possibly via single-stranded RNA present within virions that can trigger e.g. TLR7 and −8, as observed for CVB3 (**Fig. 2C**) As expected, addition of rupintrivir to poly I:C stimulated cells had no effect on ISG induction (data not shown).

### Full-blown BDCA1^+^ mDC Activation upon EV1 Infection

**Figure 3:**
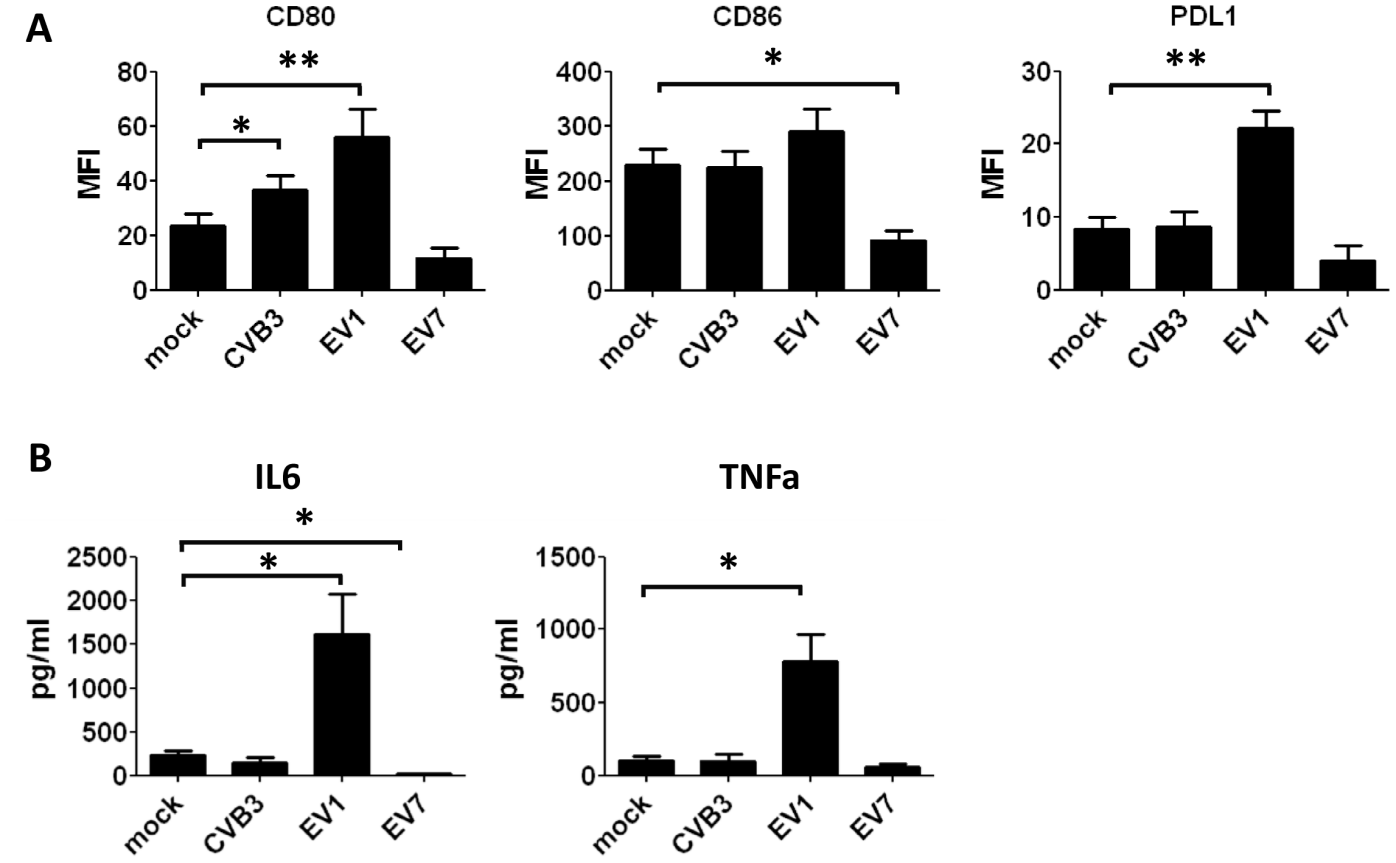
EV1 infection results in phenotypic maturation and production of IL6 and TNF-α. A) Freshly isolated BDCA1^+^ mDCs were infected as indicated (MOI 50) and after 18 h expression of cell surface markers was determined using flowcytometry. B) Supernatant taken from mDCs infected as in A) was analyzed for production of IL6 and TNF-α. Data shown (mean+SEM) are averages of at least 8 different experiments using different donors. Statistical significance determined by Students T-test, * p<0.05; **p<0.01. MFI; mean fluorescence intensity.

To investigate whether maturation is induced in BDCA1^+^ mDCs upon infection we determined expression levels of co-stimulatory (CD80 and CD86) and co-inhibitory (PDL1) molecules on their cell surface. At 18 h p.i. EV1 infection resulted in substantial induction of CD80, CD86 and PDL1 in all donors, relative to unstimulated or EV7 or CVB3 infected DCs (**Fig. 3A**). DC activation was further confirmed by IL-6 and TNF-α secretion upon EV1 infection (**Fig. 3B**). EV7-infected BDCA1^+^ mDCs showed expression lower than unstimulated mDC for all markers tested (**Fig. 3A**), correlating with the massive increase in cell death observed upon EV7 infection (**Fig. 1E** **and Fig. S6**). Also at earlier time points (e.g. 4, 8 hrs p.i.) when less cell death was apparent in EV7-infected cells, no induction of costimulatory molecules was observed (data not shown), probably because the DCs haven’t had enough time to mature. Consistent with the rapidly induced cell death, absence of costimulatory molecule induction and absence of ISG production at the protein level, EV7 infection also did not induce pro-inflammatory cytokines (**Fig. 3B**). CVB3 induced a modest, but significant increase in CD80 expression (**Fig. 3A**) in approximately half of the donors tested (n = 13); yet no CD86 or PDL1 induction was observed, and pro-inflammatory cytokines IL-6 and TNF-α were not induced upon CVB3. Upon EV1-infection, a more pronounced upregulation of CD80 and PDL1 is observed compared to CD86, probably caused by relative high expression of CD86 under unstimulated conditions (mock) that reaches a plateau-phase upon stimulation (EV1). Our data indicate that EV1 infection results in full-blown maturation of BDCA1^+^ mDCs isolated freshly from blood, whereas this is not the case upon EV7 or CVB3 infection under the conditions tested.

### EV, but Not CVB Infection Impairs TLR-induced Responses in BDCA1^+^ mDCs

**Figure 4:**
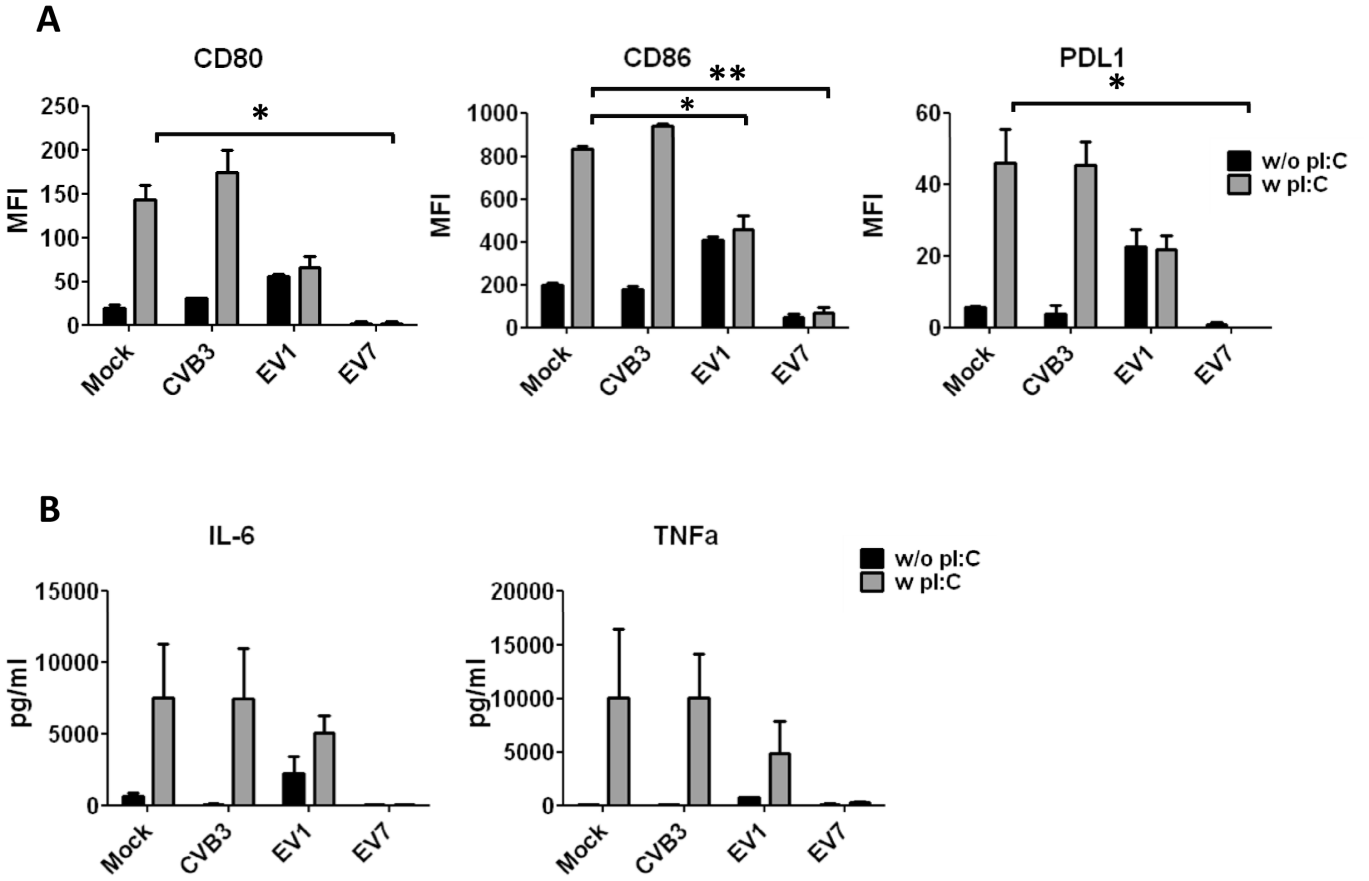
EV, but not CVB infection impairs TLR-induced responses in BDCA1+ mDCs. A) Freshly isolated BDCA1^+^ mDCs were infected as indicated (MOI 50) and after 16 h all cells were stimulated with poly I:C (20 µg/ml). After an additional 24 h expression of cell surface markers was determined using flowcytometry. Data is shown as mean fluorescent intensity minus isotype control. B) Supernatant taken from mDCs infected and stimulated as in A) was analyzed for production of IL6 and TNF-α. Shown are averages of 2 experiments using different donors. Statistical significance determined by Students T-test, * p<0.05. **p<0.01.

To analyze whether infection affects the response of mDCs to TLR ligands, they were infected with HEV-B and at 16 h p.i. stimulated with poly I:C. After 24 h poly I:C challenge co-stimulatory molecules and cytokine production were determined. Poly I:C-induced expression of CD80 and PDL1 (**Fig. 4A**), as well as IL-6 and TNF-α production (**Fig. 4B**) were not affected by CVB3 infection. In contrast, upon EV1 infection there was no further increase of CD80, CD86 or PDL1 expression compared to non poly I:C-stimulated, EV1-infected cells. Poly I:C-induced IL6 and TNF production was decreased by approximately 50% compared to mock- or CVB-infected, poly I:C-stimulated cells (**Fig. 4A and 4B**), corresponding with modest induction of cell death in BDCA1^+^ mDC. EV7 dramatically inhibited induction of cell surface markers. Furthermore, hardly any IL6 or TNF-α was induced upon poly I:C stimulation, reflecting the massive, rapid cell death that EV7 induced in BDCA1^+^ mDCs. Thus, HEV-B infection differentially affects the response of BDCA1^+^ mDC to poly I:C, and this correlates with the amount of cell death induced, suggesting that loss of viability is the main cause for lack of responses.

### BDCA1^+^ mDCs Respond More Rapidly and More Pronounced to EV1 Infection Compared to *in vitro* Differentiated moDCs

**Figure 5:**
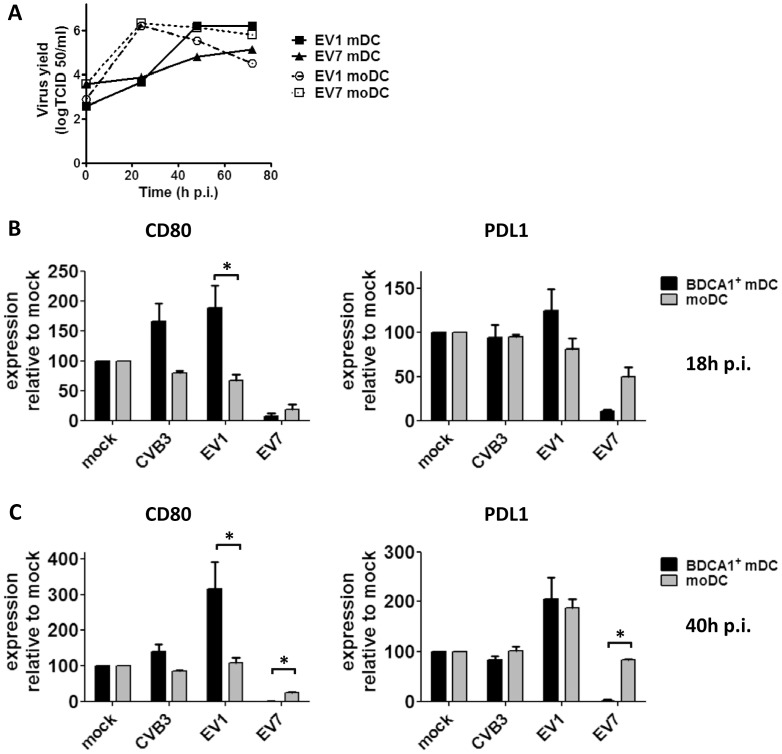
BDCA1^+^ mDC respond faster and more pronounced than moDC to EV1 infection. A) Freshly isolated BDCA1^+^ mDCs or day 6 immature moDC generated from monocytes from the same donor were infected as indicated (MOI 5) and replication was analyzed at indicated time points. B) Freshly isolated BDCA1^+^ mDCs or moDCs from the same donor were infected as indicated (MOI 50) and after 18 h cell surface marker expression was determined using flowcytometry. C) As for B) but analyzed at 40 h p.i. Shown are representative (A) or averages of 2 experiments using different donors. Expression of surface markers in mock-infected DCs is set to 100 in all subsets for comparison. Statistical significance determined by Students T-test, *P<0.05.

To relate our findings with fresh blood DCs to previous studies with *in vitro* generated moDC, we compared the replication kinetics and DC responses upon HEV-B infection. Both EV1 and EV7 replication kinetics were slower in BDCA1^+^ mDCs when using the same MOI and DCs from the same donor. Virus production increased gradually over time in BDCA1^+^ mDC cultures, whereas maximal titers were reached in moDC cultures within 12–24 hours (**Fig. 1A** **,** **Fig. 5A** **and ref** [9]), indicating that there are differences between primary BDCA1^+^ mDCs and moDCs.

CVB3 does not replicate in moDC and mDCs and no induction of CD80 or PDL1 was observed in moDC and only a modest CD80 induction in BDCA1^+^ mDCs (**Fig. 5** **B and C** and ref [9]). EV7 which replicates rapidly in moDC and, similar to EV7 infection of BDCA1^+^ mDCs, induces massive cell death, greatly reduced CD80 expression in both DC subsets. PDL1 expression in moDCs, however, showed a 50% decrease 18 h p.i., and only a very modest decrease at 40 h p.i. upon EV7 infection, whereas a nearly 100% reduction in PDL1 expression was observed in BDCA1^+^ mDCs (**Fig. 5B and C**). Comparison between EV1-infected moDC and BDCA1^+^ mDC isolated from a single donor revealed that moDC did not increase CD80 and PDL1 after overnight incubation, whereas this was the case for BDCA1^+^ mDC (**Fig. 5B**). At 40 h p.i. no increase of CD80 was observed in moDC, whereas CD80 levels further increased on BDCA1^+^ mDC. PDL1 induction was induced in moDC at 48 h p.i. and approached levels similar to those in BDCA1^+^ mDC 40 h p.i. (**Fig. 5C**). These data indicate that naturally occurring BDCA1^+^ mDC are superior compared to moDC with regard to the kinetics and the expression levels of cell surface maturation markers CD80 and PDL1 induced upon EV1 infection. In these donors, no differences in IL6 and TNFα production were observed upon EV1 infection (data not shown), confirming previous reports that induction of cytokines and upregulation of costimulatory molecules are separate processes [31], [32], since CD80 and PDL1 were differentially induced upon EV1 infection in BDCA1^+^ mDCs versus moDCs. In summary, BDCA1^+^ mDCs isolated freshly from blood respond faster and more pronounced compared to *in vitro* differentiated moDC with regard to CD80 and PDL1 induction upon EV1 infection.

## Discussion

We have previously characterized the response of *in vitro* differentiated moDCs to different HEV-Bs [9]. In this study the susceptibility and subsequent response of BDCA1^+^ mDC, which are isolated freshly from blood and thus more physiologically relevant compared to moDCs, to different HEV-B strains was assessed. The data show that HEV-Bs differentially influence human BDCA1^+^ mDCs function. EV7 efficiently infects and rapidly kills the BDCA1^+^ mDCs and as a result does not induce DC activation. In contrast, EV1 infection results in lower virus input levels, lower amounts of viral proteins and dsRNA-positive DC and modest cell death. Furthermore, EV1 infection induced ISGs which (partially) depended on virus replication and upregulated CD80, CD86 and PDL1. CVBs cannot productively infect BDCA1^+^ mDCs, but do induce modest ISG expression and CD80 expression. Additionally, we have shown that BDCA1^+^ mDCs isolated freshly from blood respond faster and more pronounced compared to *in vitro* differentiated moDC with regard to CD80 and PDL1 induction.

Major differences in BDCA1^+^ mDC viability upon EV infection were observed, with much higher cell death rates upon EV7 infection. This may relate to the expression levels of the respective virus entry receptors. VLA2 expression was low on BDCA1^+^ mDCs, correlating with less viral protein expression (3D & 3CD) and lower levels of dsRNA in EV1-infected mDCs compared to EV7. Because VLA2 expression levels are rather low, one possibility is that another, yet to be identified, receptor exists that facilitates EV1 entry into human DCs. DAF was highly expressed on mDCs, correlating with high viral protein production, high amounts of dsRNA-positive cells and massive cell death upon infection. Moreover, EV7 input levels were increased when compared to EV1 when using the same MOI, indicating that more EV7 was bound to BDCA1^+^ mDCs directly after infection. When comparing EV1 and EV7 infection on BGM cells we observed that input, replication kinetics and induction of cytopathic effects were comparable, excluding that the differences observed for BDCA1^+^ mDC are due to differences in virus titers of the inoculums or that these differences are intrinsic to EV1 and EV7 strains per se. This suggests that the expression levels of virus entry receptors on BDCA1^+^ mDCs may be responsible for the higher cell death induced by EV7 compared to EV1. Despite EV1 titers remaining a bit lower than those of EV7 during the entire replication period (data not shown), at 48 hours they reach similar values. Although indeed we did detect increased viral protein levels (3D) on western blot and more dsRNA by flowcytometry in EV7-infected cells, virus yield after 48 hours did not significantly differ from that of EV1 for reasons that are currently not well understood.

In our experiments to assess the effects on viability and function of BDCA1^+^ mDCs we have used an MOI of 50 to infect the cells. However, at lower MOIs the response of the DCs may be different, and when less virus is present the DCs may be able to respond more rapidly to e.g. EV7 infection by for example type I IFN production. Our finding that EV7 virus input levels directly after infection when using MOI 5 are comparable to the virus input levels of EV1 using MOI 50 (data not shown) may encompass such a scenario. Thus, the observed differences between EV1 and EV7 with regard to viability as well as DC maturation mainly reflect the difference in virus that is capable to initially infect BDCA1^+^ mDCs. Preliminary experiments revealed that using EV7 at lower MOI (i.e. 0.5) results in maturation of BDCA1 mDCs – although with slower kinetics compared to EV1 at MOI 50 (data not shown). When relatively low amounts of virus are infecting BDCA1^+^ mDCs, the DCs may induce an antiviral state and thereby prevent further infection and spreading of the virus and simultaneously induce DC maturation.

When comparing BDCA1^+^ mDCs isolated freshly from blood to *in vitro* differentiated moDCs we found that EV9 Hill efficiently infected moDCs from all donors tested, but that replication occurred in BDCA1^+^ mDC from only 50% of the donors, indicating that differences exist between BDCA1^+^ mDCs and moDCs. EV7 infected and rapidly killed both DC subtypes, induced no DC activation and both mDC and moDC were unable to respond to subsequent TLR-stimulation. Upon EV1 infection some upregulation of PDL1 was observed in moDC at 48 h p.i.; however, responses in BDCA1^+^ mDC appeared faster and much more pronounced. VLA2 expression levels were very low on both DC subtypes. Our data suggests that BDCA1^+^ mDCs may intrinsically be better equipped to quickly respond to EV1 infection than moDC. We and others previously reported that BDCA1^+^ mDCs express higher levels of TLR3 and TLR7 when compared to moDC [23], [33], but expression levels or RIG-I and Mda5 were similar [33]. Increased expression of these TLRs involved in sensing viral RNA may account for the more pronounced response of BDCA1^+^ mDCs compared to moDC. *In vivo*, additional mechanisms may affect susceptibility of cells to viral infection, such as low constitutive expression of type I IFNs in certain tissues, as has been described for poliovirus in mice [34].

CVB3 was not able to replicate in BDCA1^+^ mDCs from any of the donors tested; consistent with our previous studies on moDCs [9]. We cannot completely exclude that CVB infection *in vivo* may occur, since CVB-strains that infect independently of CAR have been described [35]. Additionally, CVB3 was able to infect moDCs when entry was bypassed via electroporation of CVB3 RNA directly into the cytoplasm, and we hypothesize that a similar scenario holds for BDCA1+ mDCs. Interestingly, in the absence of CVB-replication, we did observe that in approximately 70% of the donors there was induction of ISGs at the mRNA level and also CD80 was modestly upregulated in most donors. We currently don’t know what causes these differences between different donors upon CVB infection. However, our data indicate that primary BDCA1^+^ mDCs are able to “sense” CVB in the absence of measurable virus replication and subsequently respond to the virus by modest induction of the type I IFN pathway and CD80 expression. Recognition of viruses in the absence of virus replication has been described before for example for HSV, parainfluenza virus 5, Sendai virus and mumps virus [36], [37]. How BDCA1^+^ mDCs recognize and respond to CVB in the absence of measurable virus replication remains to be established.

The responses of BDCA1^+^ mDCs from different donors varied upon EV1 infection, with part (∼60%) of the donors inducing high levels of IL-6 and TNF-α, whereas the other donors responded poorly or produced no pro-inflammatory cytokines at all upon EV1 infection. The observed differences in cytokine production upon EV1 infection may reflect the use of human blood donors from the outbred human population [38]. Other studies have previously reported major inter-donor differences, for example in NK cell response [39] or with regard to Fc gamma receptor expression levels [40], and we and others have previously demonstrated that the response of pancreatic islets between different human donors to CVB differs markedly [41], [42].

When correlating our cytokine data to upregulation of CD80 and PDL1 we found that upregulation of CD80 and PDL1 is not always accompanied by production of pro-inflammatory cytokines upon EV1 infection. This suggests that induction of pro-inflammatory cytokines and induction of co-stimulatory and co-inhibitory molecules on the DC plasma membrane are differentially regulated events. This is supported by studies from Napolitani *et al.* and Krummen *et al.* who found that TLRs potently act in synergy in the induction of IL-12 and but only modestly cooperate in the induction of costimulatory molecules [31], [32], suggesting that cytokine induction and upregulation of CD80 or CD86 in DCs indeed involve separate processes.

The differences observed between different HEV-B with regard to mDC viability, activation and subsequent response to TLR ligands may have important consequences for the immune response against HEV-Bs *in vivo*. For example, rapid cell death of mDCs may hamper induction of efficient antiviral immune responses. Additionally, inhibition of antigen processing and MHC class I-dependent antigen-presentation has been reported in enterovirus-infected cells *in vitro* [43], [44]. Murine *in vivo* studies have revealed that that specific DC subsets are susceptible to CVB infection, resulting in reduced DC number as well as impaired capacity to prime CD8 T cells [45] underscoring the effects of HEV-B on DCs *in vivo*. Additionally, reduced capacity of erythroid and lymphoid progenitors to form colonies upon CVB infection has been reported in mice [46]. Yet, to our knowledge no data is available on the human *in vivo* situation that may differ markedly from the murine setting due to differences in virus tropism and the immune system in human and mice. The *in vivo* situation is complex, as DCs are exposed not only to virus particles, but also to virus-infected cells, and type I IFNs produced e.g. by HEV-B infected cells in the gastrointestinal tract, both of which can influence susceptibility of the DCs for infection. For example, we have previously shown that moDCs become protected from EV-infection when they encounter CVB-infected cells [27], [47], and speculate that this also holds true for primary mDCs. Thus the *in vivo* response of myeloid DCs in blood depends not only on the virus strain, but also on the amount of virus present and on the interplay of the DCs with surrounding (infected) tissues. Furthermore, *in vivo* other DC subsets (e.g. BDCA3^+^ mDCs or plasmacytoid (p)DCs) are present which may be less susceptible to for example EV7 infection. Additionally, cross-talk with other immune cells, such as pDCs that can produce large quantities of type I IFNs may prevent full-blown infection in BDCA1^+^ mDCs, as we have previously shown for moDCs [33]. Yet, the fact that different EVs can productively infect BDCA1^+^ blood mDCs suggests that these viruses may interfere with immune homeostasis in humans *in vivo*.

The finding that EVs are capable to infect freshly isolated BDCA1^+^ mDCs from blood opens the possibility that mDCs may serve as a reservoir for these viruses *in vivo*. Various studies have reported that HEV-B RNA can be detected in blood/PBMCs of type 1 diabetes patients [14], [48], although the source for the viral RNA remains unknown. Previous studies have shown that monocytes can be infected by HEV-B via antibody-dependent mechanisms [49], and more recently it has been described that pDCs become activated by CVB in an antibody-dependent fashion – although whether the virus also productively infects pDC was not extensively studied [50]. Our data reveal that myeloid DCs can be infected with EVs (in the absence of antiviral antibodies) and thus might be an enterovirus target *in vivo* and serve as a virus reservoir in blood. Whether, and how, this contributes to enterovirus pathogenesis, and possibly also to enterovirus-related diseases such as cardiomyopathy and type 1 diabetes remains to be established.

## Supporting Information

Figure S1 **Replication of EV9 Hill in 6 different donors.** BDCA1^+^ mDCs were infected at an MOI of 5 for 1 hour, washed to remove unbound virus and input titers (i.e. amount of virus present after 1 hour infection and subsequent washes that is bound to cells or internalized in cells within the one hour infection period) and at indicated times yield (intracellular and secreted in the supernatant combined) was determined by endpoint titration. Shown are 6 different donors. Black indicates donors in which EV9 does not replicate, filled grey symbols indicate modest replication and open grey symbols represent the donor that showed efficient replication.
(TIF)

Figure S2 **Input and yield of EV1 and EV7.** BDCA1^+^ mDCs were infected at an MOI of 10 for 1 hour, washed to remove unbound virus and input titers (i.e. amount of virus present after 1 hour infection and subsequent washes that is bound to cells or internalized in cells within the one hour infection period) and yield (intracellular and secreted in the supernatant combined) after 48 h culture were determined by endpoint titration. Shown are titers of 8 different donors+SEM. * p<0.05; ns, not significant.
(TIF)

Figure S3 **Kinetics of EV1 and EV7 on BGM cells.** BGM cells were infected at an MOI of 10 and at indicated times replication analysis was determined by endpoint titration.
(TIF)

Figure S4 **Detection of dsRNA correlates with CVB3 infection in BGM cells.** BGM cells were infected with CVB3 at indicated MOI and after 18 h infection the amount of dsRNA was assessed by intracellular dsRNA staining and analyzed by flow cytometry as described. MFI: mean fluorescence intensity of dsRNA signal.
(TIF)

Figure S5 **Replication of human EV occurs in BDCA1^+^ mDCs and not in CD19^+^ B cells.** Freshly isolated BDCA1^+^ mDC and CD19^+^ B-cells, which are depleted from PBMCs before positive selection of BDCA1^+^ mDCs, were infected as indicated (MOI 5) and replication was assessed by endpoint titration.
(TIF)

Figure S6 **Kinetics of cell viability upon EV-infection.** mDCs were infected as in Fig. 1 and at 4, 8 and 24 hours p.i. cell viability was analyzed by flowcytometry using A) Annexin V (AnnV) or B) AnnV and viability dye. The percentage of AnnV (A) or AnnV/viability dye-double positive cells (B) is shown.
(PDF)

Figure S7 **Detection of ISGs at mRNA level in EV7 infected BDCA1^+^ mDCs.** Cells were infected as in Fig. 2A and mRNA expression of RIG-I, Mda5 and OAS1 was determined 6 h p.i. by qPCR as described.
(TIF)

Figure S8 **Detection of ISGs at protein level in EV infected BDCA1^+^ mDCs.** Cells were treated as in Fig. 2B and protein expression of RIG-I and Mda5 was determined by western blotting as described.
(TIF)
