# Supplementary figures and images for: Differential Susceptibility and Response of Primary Human Myeloid BDCA1+ Dendritic Cells to Infection with Different Enteroviruses
Source: PLoS One. 2013 Apr 24;8(4):e62502. doi: 10.1371/journal.pone.0062502 (PMC3634769; doi:10.1371/journal.pone.0062502)

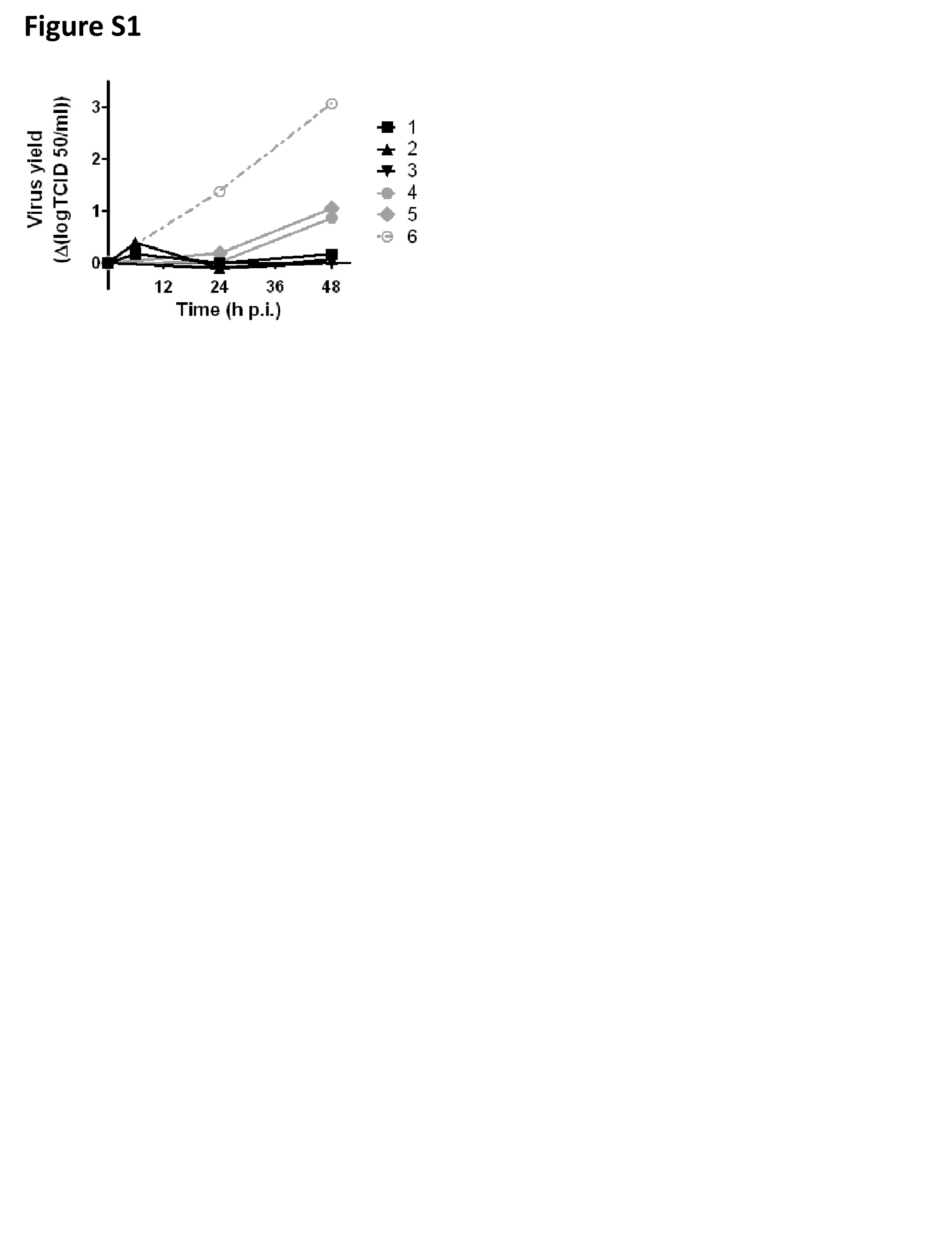

Supplement: Figure S1 — Replication of EV9 Hill in 6 different donors. BDCA1+ mDCs were infected at an MOI of 5 for 1 hour, washed to remove unbound virus and input titers (i.e. amount of virus present after 1 hour infection and subsequent washes that is bound to cells or internalized in cells within the one hour infection period) and at indicated times yield (intracellular and secreted in the supernatant combined) was determined by endpoint titration. Shown are 6 different donors. Black indicates donors in which EV9 does not replicate, filled grey symbols indicate modest replication and open grey symbols represent the donor that showed efficient replication. (TIF) [file pone.0062502.s001.tif]

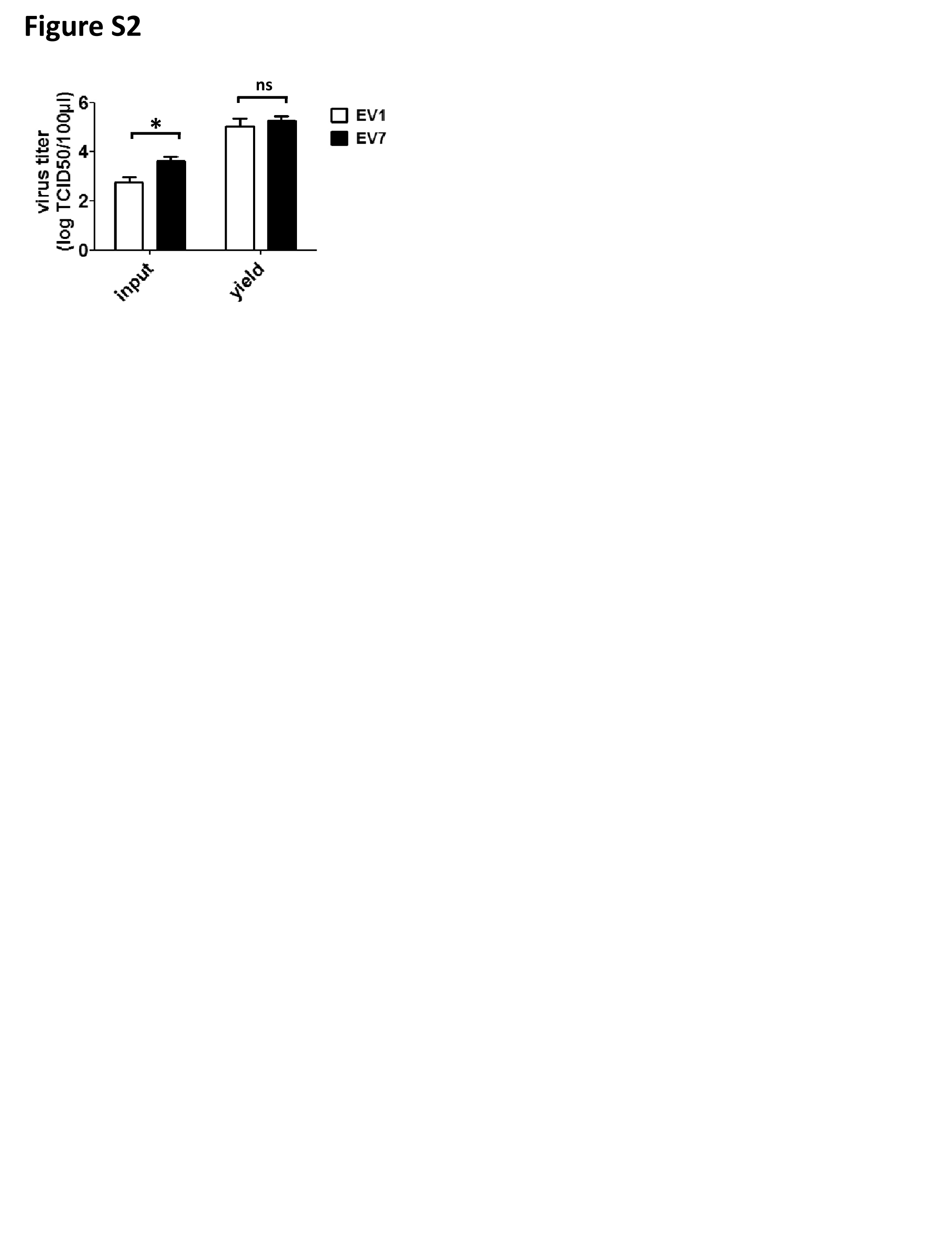

Supplement: Figure S2 — Input and yield of EV1 and EV7. BDCA1+ mDCs were infected at an MOI of 10 for 1 hour, washed to remove unbound virus and input titers (i.e. amount of virus present after 1 hour infection and subsequent washes that is bound to cells or internalized in cells within the one hour infection period) and yield (intracellular and secreted in the supernatant combined) after 48 h culture were determined by endpoint titration. Shown are titers of 8 different donors+SEM. * p<0.05; ns, not significant. (TIF) [file pone.0062502.s002.tif]

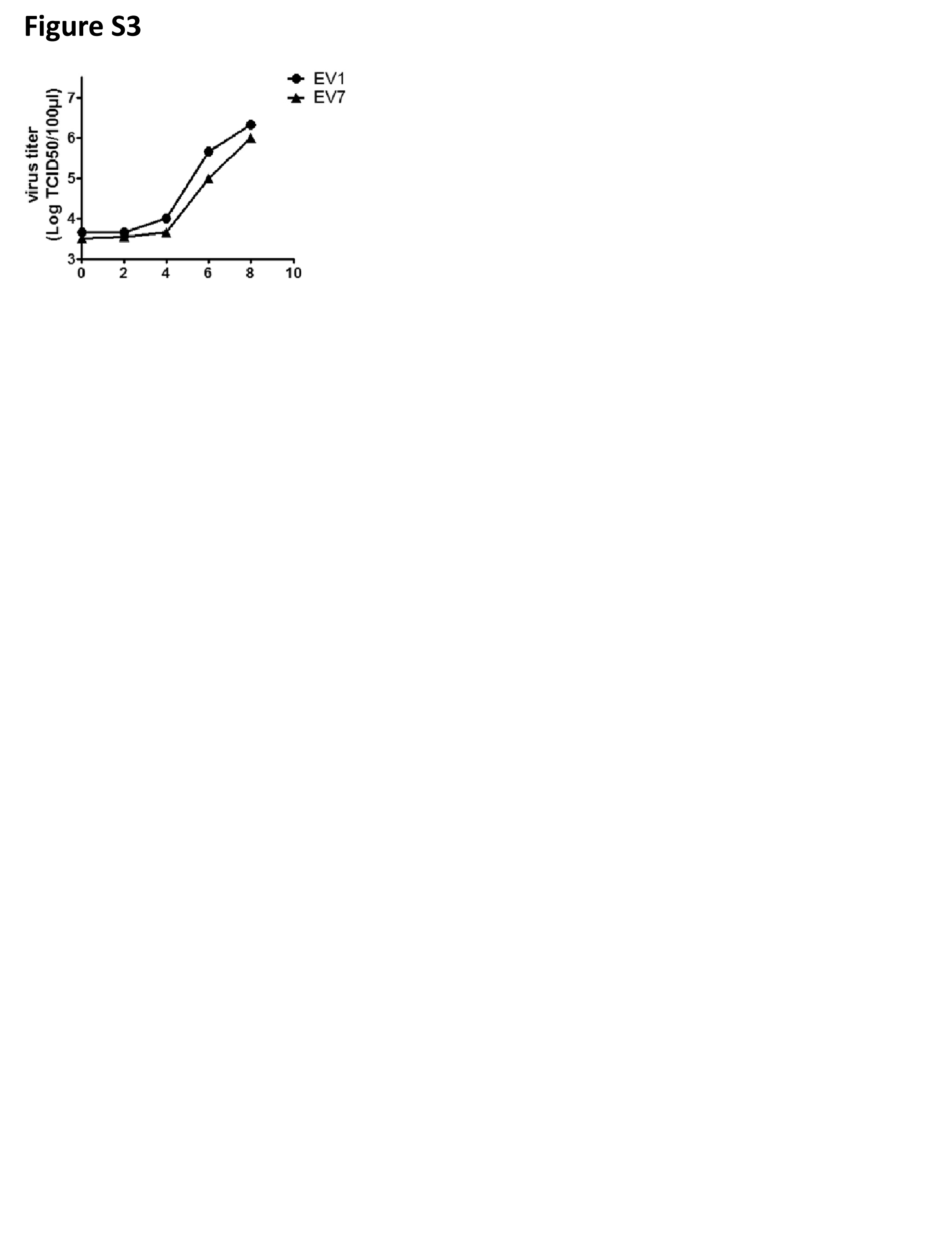

Supplement: Figure S3 — Kinetics of EV1 and EV7 on BGM cells. BGM cells were infected at an MOI of 10 and at indicated times replication analysis was determined by endpoint titration. (TIF) [file pone.0062502.s003.tif]

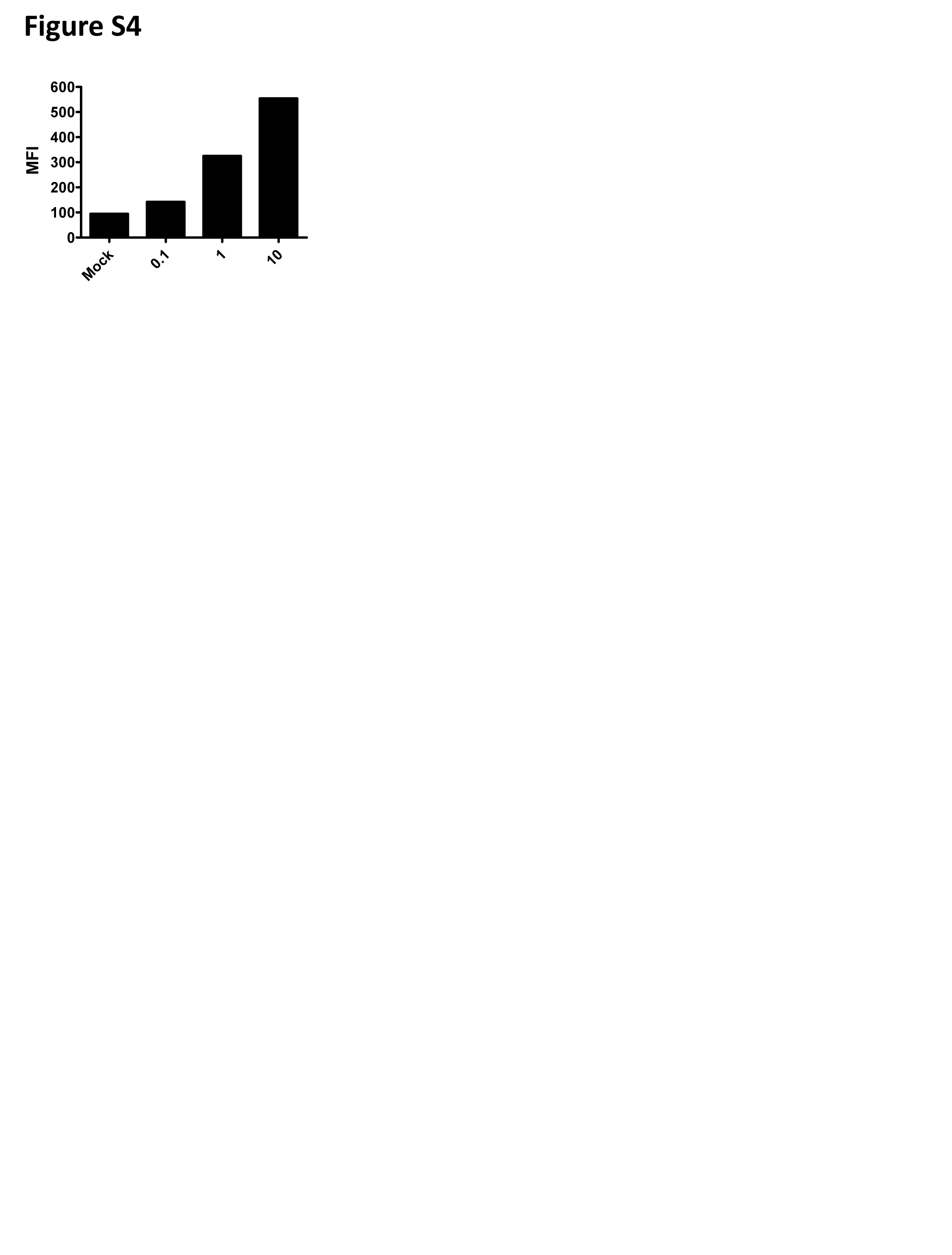

Supplement: Figure S4 — Detection of dsRNA correlates with CVB3 infection in BGM cells. BGM cells were infected with CVB3 at indicated MOI and after 18 h infection the amount of dsRNA was assessed by intracellular dsRNA staining and analyzed by flow cytometry as described. MFI: mean fluorescence intensity of dsRNA signal. (TIF) [file pone.0062502.s004.tif]

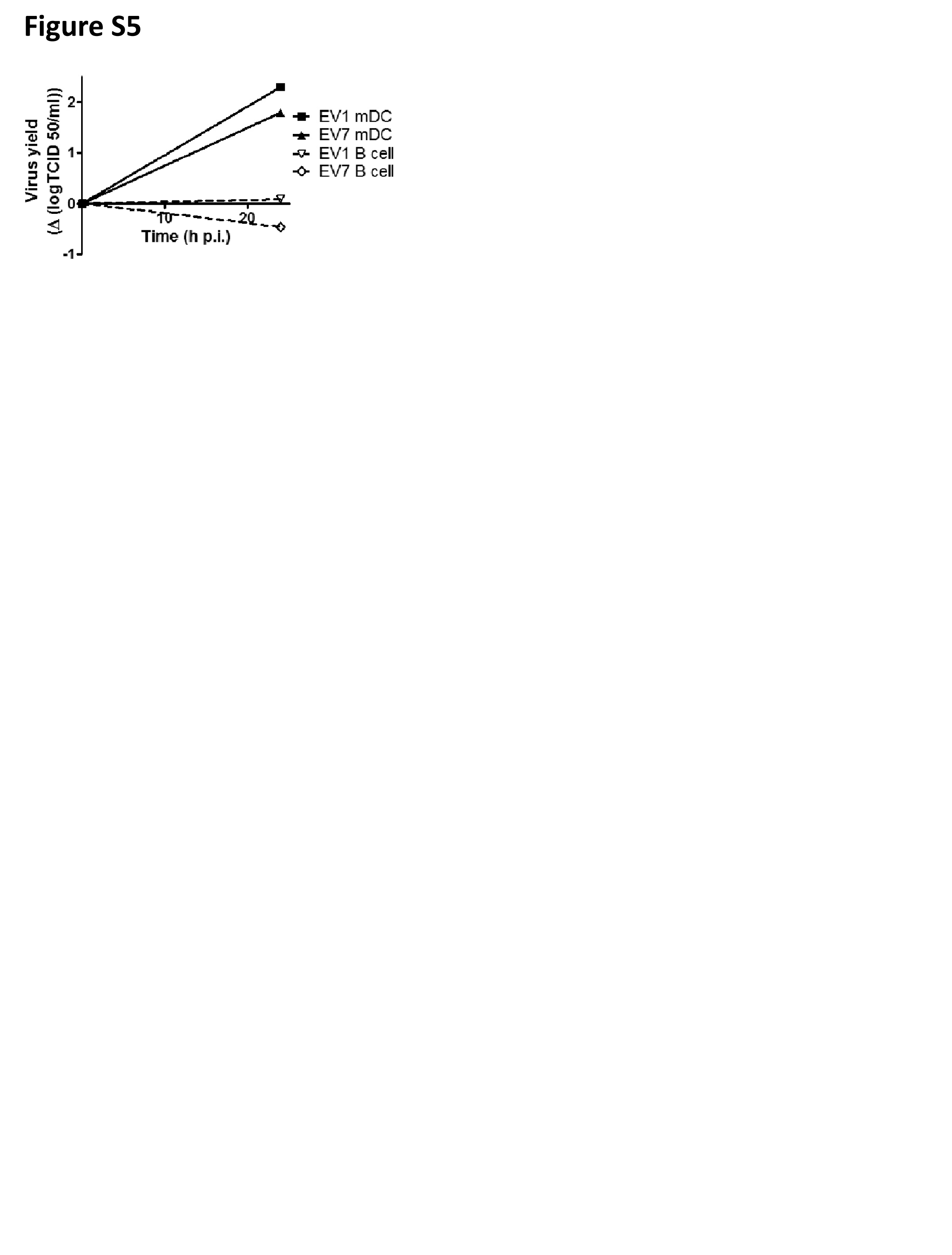

Supplement: Figure S5 — Replication of human EV occurs in BDCA1+ mDCs and not in CD19+ B cells. Freshly isolated BDCA1+ mDC and CD19+ B-cells, which are depleted from PBMCs before positive selection of BDCA1+ mDCs, were infected as indicated (MOI 5) and replication was assessed by endpoint titration. (TIF) [file pone.0062502.s005.tif]

Figure S6

S6A

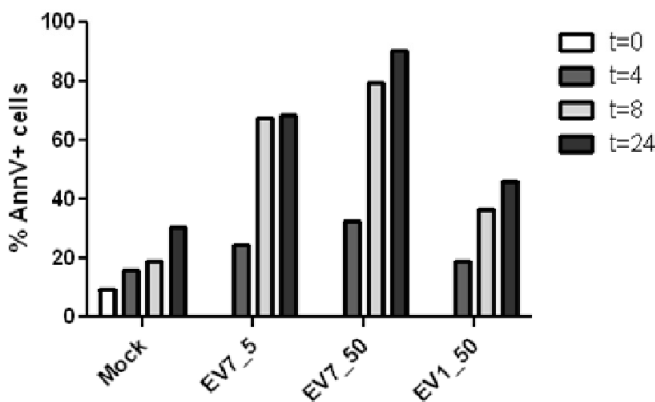

S6B

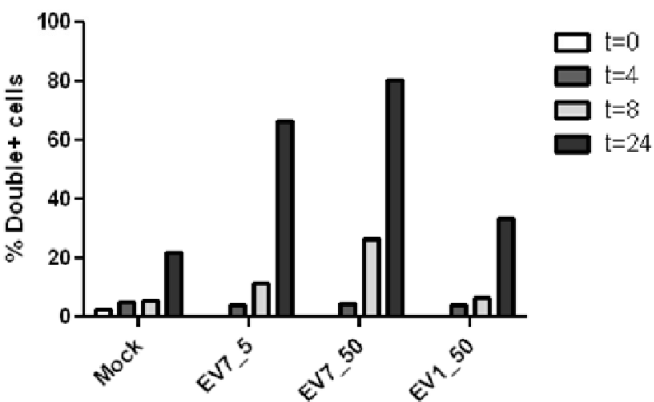

Supplement: Figure S6 — Kinetics of cell viability upon EV-infection. mDCs were infected as in Fig. 1 and at 4, 8 and 24 hours p.i. cell viability was analyzed by flowcytometry using A) Annexin V (AnnV) or B) AnnV and viability dye. The percentage of AnnV (A) or AnnV/viability dye-double positive cells (B) is shown. (PDF) [file pone.0062502.s006.pdf]

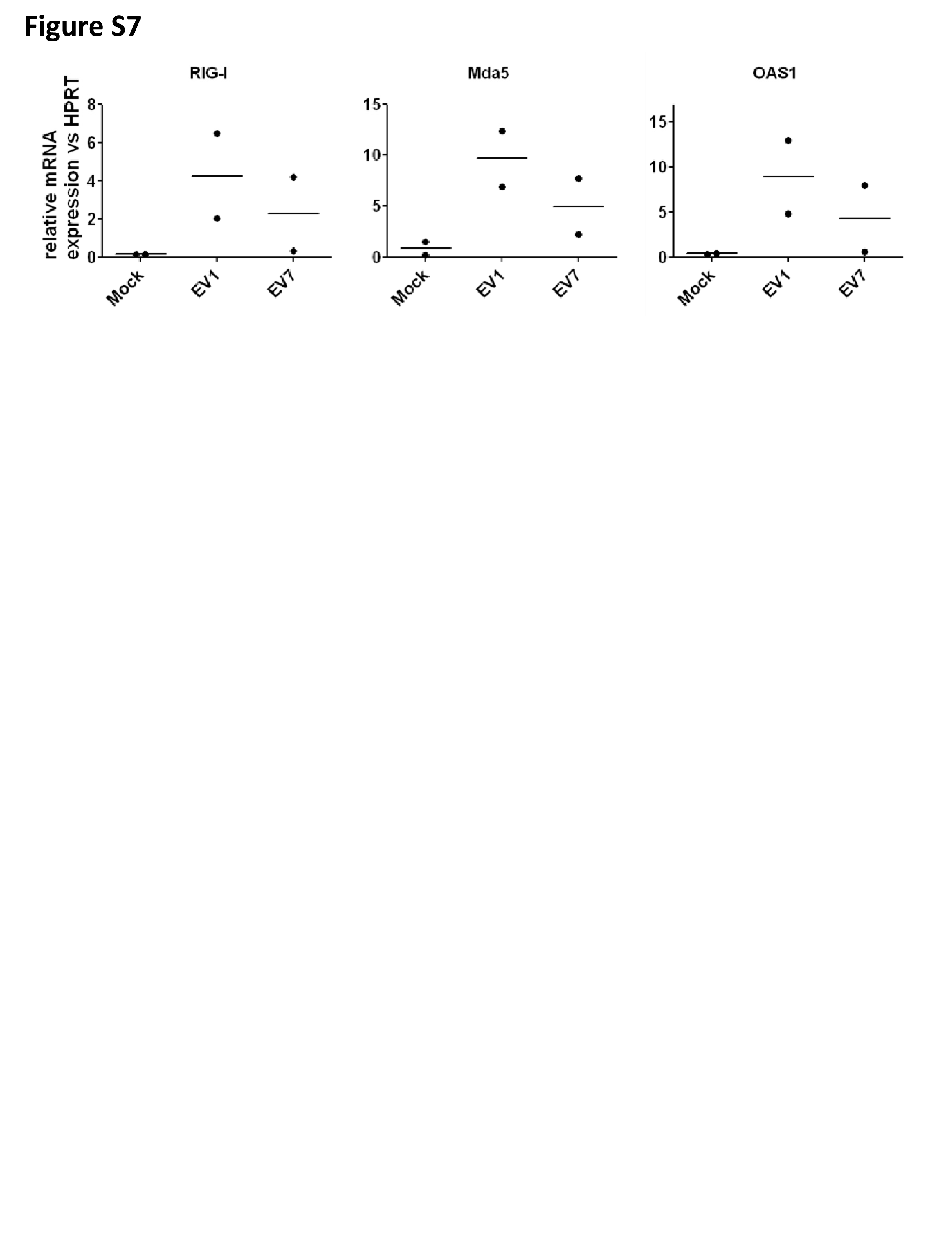

Supplement: Figure S7 — Detection of ISGs at mRNA level in EV7 infected BDCA1+ mDCs. Cells were infected as in Fig. 2A and mRNA expression of RIG-I, Mda5 and OAS1 was determined 6 h p.i. by qPCR as described. (TIF) [file pone.0062502.s007.tif]

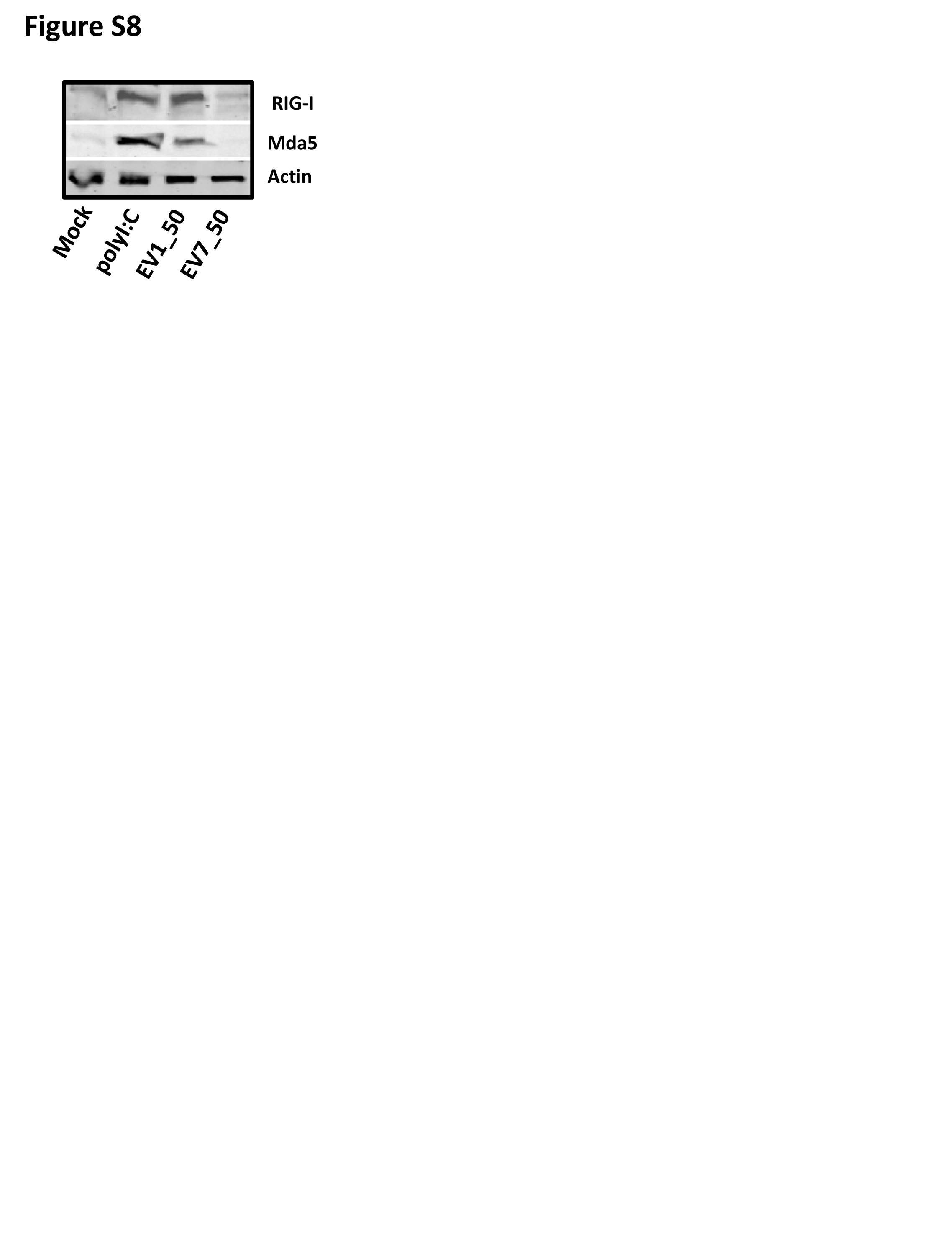

Supplement: Figure S8 — Detection of ISGs at protein level in EV infected BDCA1+ mDCs. Cells were treated as in Fig. 2B and protein expression of RIG-I and Mda5 was determined by western blotting as described. (TIF) [file pone.0062502.s008.tif]
